# Prognostic Markers in Head and Neck Cancer Patients Treated with Nivolumab

**DOI:** 10.3390/cancers10120466

**Published:** 2018-11-23

**Authors:** Daisuke Nishikawa, Hidenori Suzuki, Yusuke Koide, Shintaro Beppu, Shigenori Kadowaki, Michihiko Sone, Nobuhiro Hanai

**Affiliations:** 1 Department of Head and Neck Surgery, Aichi Cancer Center Hospital, Nagoya 464-8681, Japan; hi.suzuki@aichi-cc.jp (H.S.); kyusuke@aichi-cc.jp (Y.K.); beppin3@aichi-cc.jp (S.B.); hanai@aichi-cc.jp (N.H.); 2 Department of Clinical Oncology, Aichi Cancer Center Hospital, Nagoya 464-8681, Japan; skadowaki@aichi-cc.jp; 3 Department of Otorhinolaryngology, Nagoya University Graduate School of Medicine, Nagoya 466-8560, Japan; michsone@med.nagoya-u.ac.jp

**Keywords:** nivolumab, head and neck, squamous cell carcinoma, prognostic factor

## Abstract

To investigate whether peripheral blood biomarkers predict the outcome of anti-PD-1 antibody treatment for head and neck squamous cell carcinoma (HNSCC). Patients treated with nivolumab for platinum-refractory recurrent or metastatic HNSCC were retrospectively reviewed. Fifty-three patients treated between April 2017 and March 2018 were included in the study. The median progression-free survival (PFS) and overall survival (OS) were 2.5 and 8.7 months, respectively. In the univariate analysis, performance status (PS) ≥ 3, relative neutrophil count ≥ 0.65, relative lymphocyte count ≥ 0.17, and relative eosinophil count (REC) ≥ 0.015 were significantly associated with both PFS and OS. On multivariate analysis, PS ≥ 3 and REC ≥ 0.015 were significantly associated with PFS and OS. Low REC and poor PS were independent poor prognostic factors for both PFS and OS in patients with recurrent or metastatic HNSCC treated with nivolumab.

## 1. Introduction

Recently, the introduction of immune checkpoint inhibitors (ICIs) targeting cytotoxic T-lymphocyte antigen 4 (CTLA-4) and programmed cell death-1 (PD-1)/programmed death ligand 1 (PD-L1) has caused a breakthrough in the treatment of various cancers. CheckMate 141, a phase III randomized control study, reported that the anti-PD-1 antibody nivolumab, compared to conventional chemotherapy, improved the overall survival (OS) of platinum-refractory recurrent or metastatic head and neck squamous cell carcinoma (HNSCC) [1]. To date, nivolumab has been used as a standard second-line treatment for recurrent or metastatic HNSCC.

The definitive factors predicting treatment response and survival of head and neck cancer treated with nivolumab have not been clarified. In the CheckMate 141 study, OS was not significantly different between tumors with PD-L1 expression ≥1% and <1%. Several authors reported that baseline peripheral blood biomarkers predicted treatment outcome for several types of cancers treated with ICIs [2,3,4,5,6]. Heppt et al. reported that C-reactive protein (CRP), relative eosinophil count (REC), and lactate dehydrogenase (LDH) predicted OS of uveal melanoma treated with ICIs [2]. Recently, Ho et al. reported that pretreatment absolute lymphocyte count (ALC) was associated with progression-free survival (PFS) of recurrent or metastatic HNSCC treated with PD-1 inhibitor [7]. However, not much has been reported on the association between the outcome of anti-PD-1 antibody therapy and peripheral blood biomarkers in HNSCC. The purpose of this study was to investigate whether baseline variables, including peripheral blood biomarkers, can predict the outcome of anti-PD-1 antibody treatment for HNSCC.

## 2. Results

### 2.1. Patient Characteristics

Sixty patients were treated with nivolumab during the study period. Seven patients were excluded because one had adenocarcinoma of the submandibular gland, two did not undergo initial evaluation of treatment response because insufficient time had elapsed after nivolumab administration at the time of data collection, and four lacked PS information. As a result, 53 patients were included in this analysis. The oral cavity was the most frequent primary site, followed by the hypopharynx, oropharynx, and nasopharynx. Nivolumab injection was administered as second-line systemic therapy in 34 patients (58%) and as first-line therapy in 16 patients (27%) (Table 1). In patients treated with nivolumab as second-line or higher-line therapy, the time from last treatment was within one month. On the other hand, the median time from the last treatment was 188 days (30–1787 days) in patients who received nivolumab as first-line therapy.

### 2.2. Treatment Outcomes

**Figure 1:**
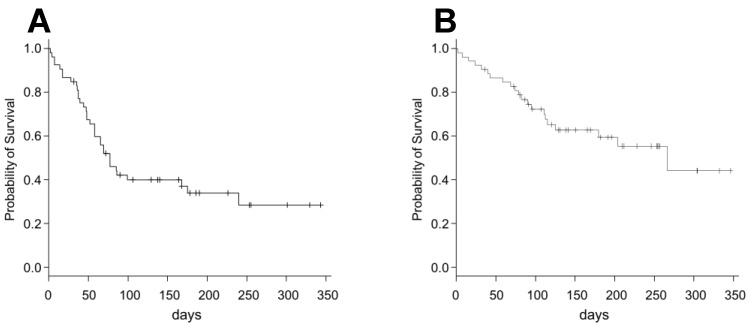
Survival curves after treatment with nivolumab in all patients: (**A**) progression-free survival; and (**B**) overall survival.

The median PFS and OS were 2.5 (95% CI 1.0–5.7) and 8.7 (95% CI 4.1–NR) months, respectively. The survival curves are shown in Figure 1. Regarding the best overall response, complete response, partial response, stable disease, and PD accounted for 3.8% (n = 2), 13.2% (n = 7), 22.6% (n = 12), and 52.8% (n = 28), respectively.

**Figure 2:**
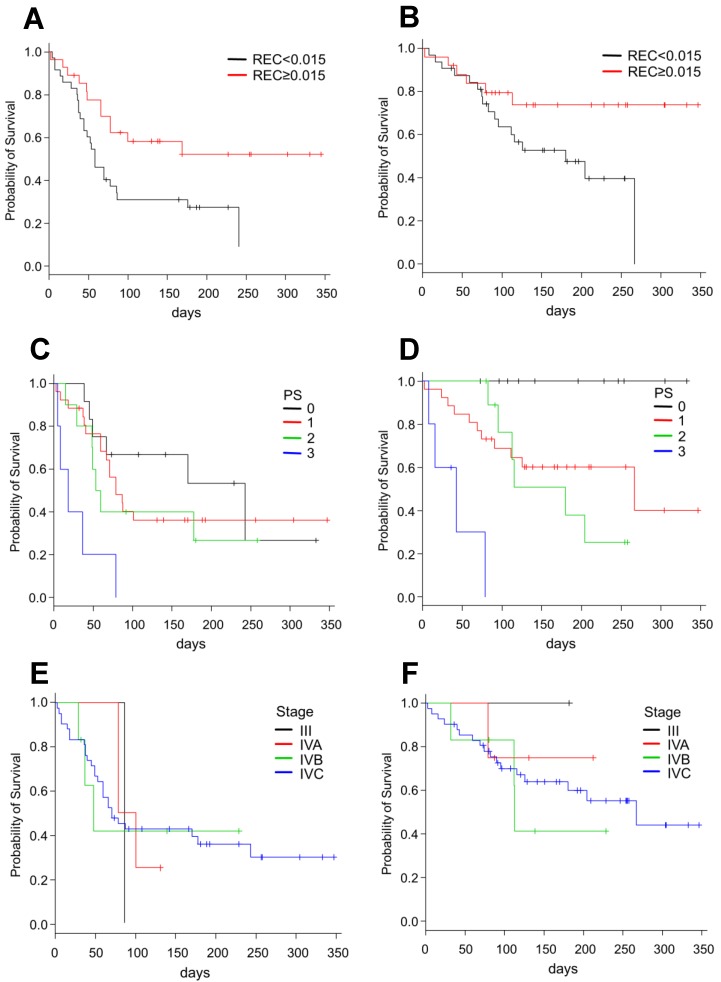
Survival curves in patients treated with nivolumab, stratified by the REC, PS and clinical stage: (**A**,**C**,**E**) progression-free survival; and (**B**,**D**,**F**) overall survival. REC, relative eosinophil count; PS, performance status.

The univariate analysis (Table 2) showed that PS ≥ 3, RNC ≥ 0.65, RLC ≥ 0.17, and REC ≥ 0.015 were significantly associated with both PFS and OS, whereas CRP ≥ 1.0 and Albumin (Alb) ≥ 3.5 were significantly associated with OS only. Conversely, LDH ≥ 240, chemotherapy line ≥ 3, and age ≥ 70 years did not significantly affect PFS or OS. No significant difference was observed between oropharyngeal cancers, which are often associated with human papilloma virus (HPV), and tumors of other anatomical regions. The Kaplan–Meier curves stratified by REC and PS are shown in Figure 2. On multivariate analysis (Table 3), PS ≥ 3 (*p* = 0.0008; HR 6.38, 95% CI 2.14–18.98) and REC ≥ 0.015 (*p* = 0.02; HR 0.31, 95% CI 0.13–0.71) were significantly associated with PFS, whereas PS ≥ 3 (*p* = 0.0002; HR 22.35, 95% CI 4.40–113.40), REC ≥ 0.015 (*p* = 0.04; HR 0.27, 95% CI 0.08–0.92), and CRP ≥ 1.0 (*p* = 0.04; HR 3.72, 95% CI 1.09–12.73) were significantly associated with OS. 

## 3. Discussion

The study results indicated some peripheral blood biomarkers are independent prognostic factors of PFS and OS in patients treated with nivolumab for recurrent or metastatic HNSCC. In particular, high CRP and low REC were the poor prognostic factors of the treatment outcomes. Furthermore, patients with poor PS achieved little benefit from nivolumab therapy.

Although a phase III randomized controlled study on patients with platinum-refractory recurrent or metastatic HNSCC showed that OS improved with nivolumab, compared to the investigator’s choice, it did not differ significantly between PD-L1 expression ≥ 1% and < 1% [1]. This result may have been due to the intratumoral heterogeneity of PD-L1 expression. Some authors reported that PD-L1’s expression rate was inconsistent between core biopsy and whole section specimens [8,9,10]. A recent study showed that a low pretreatment ALC was associated with poor PFS in HNSCC treated with PD-1 inhibitor [7]. However, other prognostic factors in HNSCC treated with ICIs have not been clarified. To our best knowledge, this study was the first to report REC as an independent prognostic factor for both PFS and OS in HNSCC treated with nivolumab. 

The study results were compatible with those for other types of cancer. Ferruci et al. reported that REC ≥ 1.5% was a favorable factor for OS in melanoma patients who received anti-CTLA-4 therapy but not chemotherapy [11]. In patients with advanced or recurrent non-small cell lung cancer (NSCLC) treated with nivolumab, high ALC (≥1000**/**mL), high absolute eosinophil count (≥150**/**mL), and low absolute neutrophil count (<7500**/**mL) significantly improved the PFS and OS [12]. Heppt et al. described that Eastern Cooperative Oncology Group (ECOG) PS, high LDH, high CRP, and REC < 1.5% were significantly associated with poor survival in metastatic uveal melanoma patients treated with either anti-PD-1 antibody monotherapy or a combination of anti-PD-1 and anti-CTLA-4 antibodies [2]. Our results were similar. 

In this study, elevated CRP was associated significantly with poor OS but not with poor PFS. McMilan et al. reported that the Glasgow prognostic score (GPS), a combination of CRP and Alb, predicted colorectal cancer prognosis [13]. Moreover, some authors reported that the GPS predicted the OS of head and neck cancer patients [14,15,16,17]. Although Chen et al. showed that the GPS was related to PFS [18], our results implied that elevated CRP had limited effects on PFS. Moreover, in our study, serum Alb had no significant relationship with treatment outcomes. The results suggested that nutritional status was less likely to affect outcomes since nivolumab therapy had few severe adverse events.

Eosinophils have direct and indirect effects on the tumor microenvironment; they release chemoattractants which induce migration of tumor-specific CD8+ T-cells to the tumor, indirectly leading to tumor elimination [19]. However, tumors become unrecognizable to cytotoxic T cells (CTLs) by losing major histocompatibility complex (MHC) class I expression on the tumor surface [20]. In this situation, Th2 cells recruit eosinophils into the tumor. Degranulating eosinophils release cytotoxic proteins, such as the major basic protein 1 and 2, and the eosinophil cationic protein, which eradicates the tumor without the MHC molecules [21,22]. Eosinophils also conduct immune surveillance of the transformed cells [23]. Eotaxin, one of the eosinophil chemokines, has been related to tumor progression in multiple cancers, such as colorectal, ovarian, and prostate cancers [24,25,26]. On the other hand, a report on eotaxin-expressing hepatocellular carcinoma showed in vivo eosinophil recruitment into tumors and infrequent tumor progression in IL-5 transgenic mice [27]. This suggested that eotaxin suppresses tumor growth in the presence of IL-5. Furthermore, eotaxin affects the immunotherapy outcome for CTL-resistant melanoma [28]. Therefore, REC may represent the strength of the immune function and influence the immunotherapy outcome.

In general, patients with poor PS have poor prognoses. Therefore, most phase III studies on systemic chemotherapy for recurrent and metastatic cancer do not include patients with poor PS. Ferreira et al. reported that PS ≥ 2 was significantly associated with a poor OS of HNSCC after treatment with cetuximab and paclitaxel [29]. For ICI, some reports showed that patients with poor PS had poor OS. In patients with advanced NSCLC treated with nivolumab, PS ≥ 2 was an independent poor prognostic factor [30]. In unresectable stage III or IV melanoma, the OS after nivolumab treatment was worse in patients with PS ≥ 1 than in those with PS = 0 [31]. In the present study, there was no significant difference between PS = 2 and PS = 1 patients. This result suggested that nivolumab administration to patients with HNSCC was rarely useful in those with PS = 3, although those with PS ≤ 2 were likely to benefit.

Some limitations of our study were its retrospective design, the short follow-up period, and the small population. In addition, the PD-L1 expression level was examined in only approximately half of the patients, and the association between p16 and the treatment outcomes has not been investigated.

## 4. Materials and Methods 

### 4.1. Patients and Treatment

This retrospective study was approved by the institutional review board of Aichi Cancer Center Hospital (2018-1-114). Patients treated with nivolumab for platinum-refractory recurrent or metastatic HNSCC between April 2017 and March 2018 at our institute were included in this study. A platinum-refractory tumor was defined as a tumor that progressed after platinum-based chemotherapy or a residual tumor after platinum-based chemoradiotherapy. We reviewed the electronic clinical records and extracted age, gender, ECOG performance status (PS), peripheral blood laboratory data, treatment history, and smoking status within one week before starting nivolumab treatment. Either a surgical or biopsy specimen was used to evaluate PD-L1 expression using immunohistochemical testing (The Dako 22C3 pharmDx; Agilent Technologies/Dako, Carpinteria, CA, USA).

The patients received nivolumab at 3 mg/kg every two weeks. Tumor response was evaluated using computed tomography imaging every 8–12 weeks, according to the Response Evaluation Criteria in Solid Tumor (RECIST) criteria version 1.1 [32]. 

### 4.2. Statistical Analysis

Statistical comparisons were two-sided, and statistical significance was defined as a *p* value of <0.05. We used the Kaplan–Meier method to plot the survival curves. Categorical variables were compared using the log-rank test. OS was defined as the time from the first nivolumab administration to the date of death or last contact. PFS was defined as the time from the first nivolumab administration to the date of progressive disease (PD) or clinically unequivocal progression. The best overall response was defined as the best response from the start of nivolumab administration to PD. The differences among groups were compared using Cox proportional hazard models. We used the area under the receiver operating characteristic curve to determine the cut-off values of continuous variables. All analyses were performed using the R version 1.6-3 software program (R Foundation for Statistical Computing, Vienna, Austria).

## 5. Conclusions

This study showed that poor PS and low REC were independent poor prognostic factors for both PFS and OS in patients with recurrent or metastatic HNSCC treated with nivolumab. Further studies including more patients and longer follow-up periods are needed.

## Figures and Tables

**Table 1:** Patient characteristics.

Characteristics		Total (N = 53)	
		N	%
Age (year)	<70	39	73.6
	≥70	14	26.4
Gender	Male	39	73.6
	Female	14	26.4
Primary site	Oral cavity	16	30.2
	Nasopharynx	8	15.1
	Oropharynx	9	17.0
	Hypopharynx	10	18.9
	Larynx	4	7.6
	Other	6	11.3
ECOG PS	0	12	22.6
	1	26	49.1
	2	10	18.9
	3	5	9.4
Clinical stage (AJCC)	III	1	1.9
	IVA	3	5.7
	IVB	6	11.3
	IVC	43	81.1
Chemotherapy line	1	13	24.5
	2	32	60.4
	≥3	8	15.1
PD-L1 expression	<1%	4	7.6
	1–49%	13	24.5
	≥50%	4	7.6
	Unknown	32	60.4
Radiation history	Yes	46	86.8
	No	7	13.2
Previous systemic therapy regimen	Platinum-based	39	73.6
	Taxane-based	15	28.3
	Cmab-contained	23	43.4
	other	3	5.7

ECOG, Eastern Cooperative Oncology Group; PS, performance status; PD-L1, programmed death ligand 1; AJCC, American Joint Committee on Cancer; Cmab, cetuximab.

**Table 2:** Univariate analysis of PFS and OS.

Variables		PFS			OS		
		HR	95% CI	*p* Value	HR	95% CI	*p* Value
Age (years)	≥70 vs. <70	0.80	0.36–1.75	0.58	1.57	0.64–3.83	0.32
Gender	Female vs. Male	1.98	0.99–3.95	0.05	1.37	0.56–3.37	0.48
ECOG PS	≥3 vs. 0–2	5.07	1.89–13.6	0.001	11.58	3.23–41.44	0.0001
Smoking status	Smoker vs. never	0.62	0.31–1.23	0.17	0.62	0.26–1.48	0.28
Primary site	Oropharynx vs. Other	0.91	0.37–2.19	0.83	0.47	0.11–2.07	0.32
Clinical stage (AJCC)	IVC vs. III, IVA, IVB	0.98	0.42-2.26	0.96	1.04	0.35-3.12	0.94
Albumin (mg/dL)	≥3.5 vs. <3.5	0.95	0.46–1.97	0.90	0.41	0.18–0.95	0.03
CRP (mg/dL)	≥1.0 vs. <1.0	1.36	0.71–2.58	0.34	3.40	1.34–8.65	0.009
LDH (IU/L)	≥240 vs. <240	1.25	0.57–2.78	0.57	0.85	0.29–2.51	0.77
RNC	≥0.65 vs. <0.65	3.61	1.39–9.34	0.008	11.01	1.48–81.92	0.01
RLC	≥0.17 vs. 0.17	0.45	0.21–0.95	0.03	0.21	0.06–0.73	0.01
REC	≥0.015 vs. <0.015	0.41	0.20–0.82	0.01	0.38	0.15–0.99	0.04
Chemotherapy line	≥3 vs. 1–2	0.56	0.20–1.59	0.28	1.51	0.55–4.08	0.41
Cmab history	Yes vs. No	1.01	0.53–1.92	0.95	1.36	0.59–3.12	0.45
PD-L1 expression (%)	≥1 vs. 1<	0.65	0.17–2.44	0.53	0.36	0.06–2.16	0.26

ECOG, Eastern Cooperative Oncology Group; PS, performance status; CRP, C-reactive protein; LDH, lactate dehydrogenase; RNC, relative neutrophil count; RLC, relative lymphocyte count; REC, relative eosinophil count; Cmab, cetuximab; PD-L1, programmed death ligand 1; AJCC, American Joint Committee on Cancer.

**Table 3:** Multivariate analysis of PFS and OS.

Variable		PFS			OS		
		HR	95% CI	*p* Value	HR	95% CI	*p* Value
Gender	Female vs. Male	2.00	0.95–4.21	0.06			
ECOG PS	≥3 vs. 0–2	6.38	2.14–18.98	0.0008	22.35	4.40–113.40	0.0002
Albumin (mg/dL)	≥3.5 vs. <3.5				0.82	0.29–2.32	0.65
CRP (mg/dL)	≥1.0 vs. <1.0				3.72	1.09–12.73	0.04
RNC	≥0.65 vs. <0.65	2.60	0.62–10.93	0.19	5.26	0.37–74.25	0.22
RLC	≥0.17 vs. 0.17	1.33	0.41–4.34	0.62	1.16	0.15–9.14	0.86
REC	≥0.015 vs. <0.015	0.31	0.13–0.71	0.005	0.27	0.08–0.92	0.04

ECOG, Eastern Cooperative Oncology Group; PS, performance status; CRP, C-reactive protein; RNC, relative neutrophil count; RLC, relative lymphocyte count; REC, relative eosinophil count.
